# PvdQ Quorum Quenching Acylase Attenuates *Pseudomonas aeruginosa* Virulence in a Mouse Model of Pulmonary Infection

**DOI:** 10.3389/fcimb.2018.00119

**Published:** 2018-04-26

**Authors:** Putri D. Utari, Rita Setroikromo, Barbro N. Melgert, Wim J. Quax

**Affiliations:** ^1^Department of Chemical and Pharmaceutical Biology, University of Groningen, Groningen, Netherlands; ^2^Department of Pharmacokinetics, Toxicology and Targeting, University of Groningen, Groningen, Netherlands

**Keywords:** *Pseudomonas aeruginosa*, PvdQ acylase, quorum sensing, quorum quenching, mouse model, pulmonary infection

## Abstract

*Pseudomonas aeruginosa* is the predominant pathogen in pulmonary infections associated with cystic fibrosis. Quorum sensing (QS) systems regulate the production of virulence factors and play an important role in the establishment of successful *P. aeruginosa* infections. Inhibition of the QS system (termed quorum quenching) renders the bacteria avirulent thus serving as an alternative approach in the development of novel antibiotics. Quorum quenching in Gram negative bacteria can be achieved by preventing the accumulation of *N*-acyl homoserine lactone (AHL) signaling molecule via enzymatic degradation. Previous work by us has shown that PvdQ acylase hydrolyzes AHL signaling molecules irreversibly, thereby inhibiting QS in *P. aeruginosa in vitro* and in a *Caenorhabditis elegans* model of *P. aeruginosa* infection. The aim of the present study is to assess the therapeutic efficacy of intranasally instilled PvdQ acylase in a mouse model of pulmonary *P. aeruginosa* infection. First, we evaluated the deposition pattern of intranasally administered fluorochrome-tagged PvdQ (PvdQ-VT) in mice at different stages of pulmonary infection by *in vivo* imaging studies. Following intranasal instillation, PvdQ-VT could be traced in all lung lobes with 42 ± 7.5% of the delivered dose being deposited at 0 h post-bacterial-infection, and 34 ± 5.2% at 72 h post bacterial-infection. We then treated mice with PvdQ during lethal *P. aeruginosa* pulmonary infection and that resulted in a 5-fold reduction of lung bacterial load and a prolonged survival of the infected animals with the median survival time of 57 hin comparison to 42 h for the PBS-treated group. In a sublethal *P. aeruginosa* pulmonary infection, PvdQ treatment resulted in less lung inflammation as well as decrease of CXCL2 and TNF-α levels at 24 h post-bacterial-infection by 15 and 20%, respectively. In conclusion, our study has shown therapeutic efficacy of PvdQ acylase as a quorum quenching agent during *P. aeruginosa* infection.

## Introduction

*Pseudomonas aeruginosa* is an opportunistic Gram negative bacterium that is mainly associated with hospital-acquired infections and known as the major pathogen in cystic fibrosis (CF) patients (Driscoll et al., 2007). Nearly all pulmonary *P. aeruginosa* infections in CF patients will develop into chronic, persistent infections that require aggressive antibiotic treatments (Van Delden and Iglewski, 1998). The intrinsic traits of this bacterium coupled with complex adaptive behaviors such as biofilm formation make it resilient to many antibiotic treatments (Breidenstein et al., 2011). All of these elements propelled *P. aeruginosa* into a significant multidrug-resistant pathogen worldwide.

In numerous pathogens, production of bacterial virulence determinants is tightly regulated in a cell density-dependent manner, aided by a quorum sensing (QS) signaling system (Fuqua and Greenberg, 2002). By detecting the accumulation of signal molecules, each individual cell is capable of sensing the population density and subsequently responds by producing an arsenal of virulence factors when a critical population mass is reached (Cámara et al., 2002). The most studied signaling molecules in Gram-negative bacteria are *N*-acyl homoserine lactones (AHLs) (Papenfort and Bassler, 2016). The AHLs are produced by AHL-synthases (e.g., LuxI-type family) and sensed by transcriptional regulators (LuxR-type family) (Fuqua and Greenberg, 2002). The core of QS in *P. aeruginosa* consists of two LuxRI*-*based signaling systems that work in a hierarchal fashion, namely LasRI and RhlRI with 3-oxo-C12-HSL and C4-HSL as their respective cognate AHL (Jimenez et al., 2012). Deletion of either the AHL synthases or AHL receptors resulted in a downregulation of QS-regulated virulence factors, such as rhamnolipids, elastase protease, pyocyanin siderophore, and biofilm formation (Passador et al., 1993; Whiteley et al., 1999). These QS mutants are less pathogenic in animal models in comparison to the wild-type (Wu et al., 2001; Imamura et al., 2005), revealing the importance of this system for establishing successful infections. These findings opened up a possibility of attacking QS system as a new antivirulence drug therapy.

Quorum sensing (QS) inhibition (termed quorum quenching, QQ) can be performed by employing small molecule inhibitors to block the AHL productions or to avoid the interaction between AHLs and the response regulators. Bioactive compounds isolated from natural sources, or ones that are synthetized chemically, have shown therapeutic efficacy as QS inhibitors (QSIs) both *in vitro* and *in vivo* (Hentzer et al., 2002; Bjarnsholt et al., 2005; Rasmussen et al., 2005; Jakobsen et al., 2012a,b). However, some well-known small molecules inhibitors (QSIs), such as patulin and furanones, are toxic for mammals (Hentzer and Givskov, 2003; Puel et al., 2010) diminishing their potential for use humans. Another obvious approach for QS inhibition is by preventing accumulation of signal molecules by means of enzymatic degradation (Kalia, 2013). So far, three classes of enzymes have been identified that are known to inactivate AHLs, namely (i) AHL-lactonases [that cleave the ester bond in the homoserine lactone (HSL) ring moiety; Dong et al., 2000; Wang et al., 2010], (ii) AHL-acylases (that irreversibly hydrolyze the amide bond between the acyl chain and HSL; LaSarre and Federle, 2013), and the least studied (iii) AHL-oxidoreductases (that modify the 3-oxo-substituents of the AHLs; Uroz et al., 2005).

Numerous AHL-inactivating enzymes (QQ enzymes) were characterized, but only lactonase has been tested for its efficacy in mammalian models of pulmonary infection (Migiyama et al., 2013; Hraiech et al., 2014). Due to the large size of the enzyme molecules, the only possible route of administration is via the upper respiratory tract. Combining the procedures of establishing the infection and delivering the drug via the upper respiratory tract is challenging to be performed in small animals. Therefore, the recent study on the administration of an AHL-lactonase was done in rats using intubation of trachea. It successfully reduced mortality in the rat model of pneumonia (Hraiech et al., 2014). However, there is yet no study that employs a non-invasive drug administration method that closely mimics the actual procedure in human.

The purpose of our study was to determine the efficacy of one of the other AHL-inactivating enzymes, an AHL-acylase that was instilled intranasally in a mouse model of pulmonary *P. aeruginosa* infection. Our enzyme of interest is PvdQ acylase, a periplasmic enzyme from *P. aeruginosa* that is suggested to be involved in the maturation of pyoverdine siderophore (Drake and Gulick, 2011). Beside this function, PvdQ is a well-studied AHL-hydrolyzing enzyme, with specificity to long chain AHLs (Sio et al., 2006; Bokhove et al., 2010). PvdQ, either overexpressed in, or exogenously supplemented to *P. aeruginosa*, could significantly attenuate the virulence production, both *in vitro* (Sio et al., 2006), and *in vivo* in a *Caenorhabditis elegans* model (Papaioannou et al., 2009). In this report, we show results of PvdQ acylase deposition in the respiratory tract after intranasal administration and its efficacy in lethal and sublethal models of pulmonary *P. aeruginosa* infection.

## Materials and methods

### Bacterial strains and growing condition

Enzymatic hydrolysis of long chain AHL was monitored by employing a reporter strain *E. coli* pSB1075 (Amp^R^) (Winson et al., 1998). Determination of PvdQ inhibition strength was performed by reporter strains *P. aeruginosa PlasB::lux* (Koch et al., 2014) and *PrhlA::lux* (Tet^R^) (this study). *P. aeruginosa* PAO1 was obtained from Barbara Iglewski (University of Rochester Medical Center, Rochester, NY) (Sio et al., 2006). The overnight cultures of the biosensors were prepared by inoculating a loop of frozen glycerol stock in Luria Bertani (LB) medium, followed by incubation at 37°C, 200 rpm. For the animal experiments, *P. aeruginosa* PAO1 from a frozen glycerol stock was grown in *Pseudomonas* isolation agar (PIA) selection medium (BD Difco™) overnight at 37°C. A single colony was used to inoculate a 100 mL LB medium in a 250 mL erlenmeyer flask, at 37°C, 200 rpm for 18 h. When necessary, 100 μL/mL tetracycline or 50 μL/mL ampicillin was added to the media.

### Preparation of PvdQ

#### Production and purification of PvdQ

PvdQ was produced and purified as reported previously (Bokhove et al., 2010), with modifications. *E. coli* DH10B harboring pMCT_*pvdQ* was grown in 2xTY medium with chloramphenicol supplementation (50 μg/mL) for 30 h at 30°C, 200 rpm. The harvested cells were sonicated in a three times volume of lysis buffer (50 mM Tris Cl pH 8.8; 2 mM EDTA), followed by centrifugation at 17.000 rpm for 1 h. The clear lysate was applied to an anion exchange HiTrap Q-sepharose column and the flowthrough containing PvdQ was collected. After adjusting the ammonium sulfate concentration to 750 mM, the solution containing PvdQ was applied to a phenyl sepharose column. PvdQ eluted at the end of the 1,000–0 mM ammonium sulfate gradient. The buffer was exchanged into 50 mM sodium phosphate pH 6.5 and the sample was applied to a HiTrap Q-sepharose column. The collected flowthrough was subsequently concentrated and applied to a gel filtration superdex 16/60 75. A major peak containing PvdQ was collected, snap frozen and stored at −80°C until further use. All protein chromatography columns were obtained from GE Healthcare Life Sciences.

#### Endotoxin removal from the purified PvdQ

For animal experiments, endotoxin contamination in purified PvdQ was eliminated using a Pierce™ High Capacity Endotoxin Removal Resin (Thermo Scientific) following the manufacturer's manual. To adjust the PvdQ concentration, an endotoxin-free PBS buffer (Millipore, Merck) was used. The endotoxin content of purified PvdQ was analyzed with the LAL test at the University Medical Center Groningen, the Netherlands.

#### Fluorochrome labeling of PvdQ

For the purpose of the PvdQ deposition study in mice, PvdQ was labeled with VivoTag 680 XL Fluorochrome (Perkin Elmers). 0.5 mg PvdQ (1 mg/mL) was labeled according to the manufacturer's manual. The calculated degree of labeling was 2, indicating that in average 2 dye molecules were coupled to one molecule of PvdQ.

### *In vitro* quorum quenching activity of PvdQ

#### Enzymatic activity of PvdQ in hydrolyzing 3-oxo-C12-HSL

The enzymatic activity of PvdQ in deacylating 3-oxo-C12-HSL (Cayman Chemical) was validated using a bioassay procedure as previously described (Wahjudi et al., 2011). *E. coli* JM109 (pSB1075) biosensor that emits luminescence in the presence of long-chain AHLs was employed to detect the remaining 3-oxo-C12-HSL. Briefly, 2 μL of 0.5 mg/mL 3-oxo-C12-HSL in acetonitrile was spotted onto a flat-bottom μClear white microplate (Greiner Bio-One) and incubated at the room temperature until the acetonitrile evaporated. The remaining AHL was solubilized in 100 μL PBS buffer pH 7.4 containing 5 μg of PvdQ. A control reaction was prepared in identical conditions using heat-inactivated PvdQ. After 4 h at 30°C with slow agitation, 100 μL of the 100 times diluted overnight biosensor was added to each well. The emitted luminescence and the bacterial growth (OD_600_) were monitored in a FLUOstar Omega platereader (BMG Labtech).

#### Quorum quenching activity of PvdQ in *P. aeruginosa* reporter strains

The following assays were performed to determine the quorum sensing inhibition activity of PvdQ by employing *P. aeruginosa* biosensors. *P. aeruginosa* P*rhlA::lux* and P*lasB::lux* each containing a chromosomal insertion of a luciferase gene under the control of a *rhlA* rhamnolipid promoter or a *lasB* elastase promoter, respectively (Koch et al., 2014). Two-fold serial dilutions of PvdQ in PBS (100 μL) were made in a flat-bottom μClear white microplate (Greiner Bio-One), covering PvdQ concentration of 0–16 μM. Overnight cultures of the biosensors were diluted 100 times in LB, and 100 μL was added to the wells containing PvdQ. The emitted luminescence and the bacterial growth (OD_600_) were monitored in a FLUOstar Omega platereader (BMG Labtech).

### Epithelial cell viability assay

The effect of PvdQ on the cell viability was assessed in the lung epithelial cell lines A549 and H460. Serial 2-fold dilutions of PvdQ with a maximum concentration of 10 μM were added to 10^5^ cells, followed by incubation at 37°C for 48 h. The level of cell proliferation was determined by a 3-(4,5-dimethylthiazol-2-yl)-5-(3-carboxymethoxyphenyl)-2-(4-sulfophenyl)-2H-tetrazolium (MTS salt, Promega) proliferation assay according to the manufacturer's manual.

### Preparation of the agarose-embedded bacteria

One day prior to infection of animals, *P. aeruginosa* PAO1 was embedded in agarose as explained elsewhere (van Heeckeren and Schluchter, 2002; Kukavica-Ibrulj et al., 2014), with modifications. Cell pellets from 100 mL overnight culture of *P. aeruginosa* PAO1 were washed twice with a sterile PBS, and were resuspended in 5 mL LB. A volume of 1 mL bacterial suspension was added to 10 mL 1.5% sterilized, pre-warmed (48–50°C) agarose (Type I Low EEO, Sigma-Aldrich) and mixed thoroughly. To prepare sterile agarose beads, a sterile LB medium was added to the agarose solution. The mixture was pipetted dropwise into the center of stirred vegetable oil (200 mL) that was equilibrated at ~50°C. The stirring was kept at 1500 rpm for 6 min at ~50°C. Afterwards, the emulsion was stirred slowly at 4°C for 20 min, followed by incubation on ice for 20 min. 100 mL oil in the top layer was discarded, and the remaining agarose beads were washed with PBS, followed by centrifugation in a swinging bucket rotor at 2,700 × *g*, 4°C for 15 min. The beads were subsequently washed one time with 0.5% sodium deoxycholic acid (SDC, Sigma-Aldrich) in PBS, one time with 0.25% SDC, and 4 times with PBS. After the last wash, PBS was added to the agar beads in a ratio of 2:1. The agarose beads slurry was stored at 4°C prior to use the following day. A homogenized aliquot of the agarose beads was serially diluted and plated onto PIA medium, followed by incubation at 37°C for 24 h. Based on the counted colony forming unit (CFU) on PIA plates, the original agarose beads slurry was adjusted with PBS to 1.25 × 10^7^ CFU/mL (lethal dose) or 6.25 × 10^6^ CFU/mL (sublethal dose) and 40 μL of the bacterial preparation was administered per animal.

### Animal experiments

Animal experiments were conducted in accordance with the Dutch Animal Protection Act and were approved by the Netherlands National Committee for the protection of animals used for scientific purposes (DEC6692, AVD105002017854). The experiments were performed in a BSL-2 area in the Central Animal Facility (CDP) of the University Medical Center Groningen (UMCG). Female BALB/c mice aged 11–12 weeks old with a minimum weight of 20 grams (at the start of experiment) were purchased from Charles River, France. Groups of 4–6 mice were housed in individual ventilator cages with unrestricted access to food and water. Infected animals were placed in cages with warming pads at the bottom of the cage.

### Infection procedure and intranasal PvdQ administration

The procedure for developing pulmonary infection in our study was a combination between intratracheal instillation of bacteria at the start of the experiment, and a daily intranasal delivery of the drug.

#### Intratracheal instillation of bacteria

Sterile agarose beads or agarose beads laden with *P. aeruginosa* PAO1 were instilled into the lungs via nonsurgical intratracheal administration (Bivas-Benita et al., 2005; Munder and Tümmler, 2014). Mice were anesthetized by isoflurane inhalation and the depth of anesthesia was checked by the foot reflex toward pinching. Mice were then placed vertically by the upper teeth on an intubation stand, with continuous anesthesia through a nose cone. Cold light was placed in front of the throat and the tongue was retracted to the side using forceps. When the trachea was visualized, a disposable sterile intravenous G20 catheter (BD Insyte-W) with an adjusted length was inserted into the trachea. To confirm that the catheter was indeed inside the trachea, a ventilator (Harvard Minivent) was connected. Correct catheter placement will show the chest, but not the abdomen, moving in synch with the ventilator's programmed rate. Afterwards, 40 μL of agarose beads were carefully administered into the catheter, followed by blowing 200–400 μL of air into the catheter to ensure that all beads were delivered into the lungs. While the animal was still under anesthesia, a transponder microchip (IPTT-300, BMDS) for temperature measurement was implanted subcutaneously. This transponder allows body temperature measurement with a portable reader device (DAS-7006s, BMDS) by scanning the mice without direct contact. The mice were weighed daily and their general appearance (body temperature, coat condition, behavior and locomotion) was monitored 2–3 times a day. At designated time points, the mice were anesthetized with isoflurane and euthanized by cardiac exsanguination. Blood, bronchoalveolar lavage fluid, kidney, spleen, and lungs were collected aseptically from the animals.

#### Intranasal PvdQ administration

Mice were lightly anesthetized with isoflurane and held in a ~60° inclined supine position. Subsequently, 50 μL PvdQ was instilled dropwise onto the nose of the anesthetized animal. The control group (PBS-treated) received an intranasal administration of 50 μL PBS.

### Study design

The *in vivo* study consisted of three parts: Study 1. Mouse tolerance of PvdQ; Study 2. *In vivo* imaging to monitor deposition of intranasally administered PvdQ; Study 3. An efficacy study of PvdQ in a mouse pulmonary infection model.

#### Study 1. mouse tolerance of the intranasally administered PvdQ

To determine tolerance of PvdQ, groups of mice were intratracheally challenged with sterile agarose beads and received a daily intranasal administration of PvdQ (25 and 250 ng/g body weight) or PBS. Animals from each group was sacrificed at 24, 48, or 72 h after the first intranasal administration for analysis of immune responses or inflammation. Experiments were performed in duplicate, totaling to 4 animals per group.

#### Study 2. *in vivo* imaging to monitor deposition of intranasally administered PvdQ

As PvdQ is a protein, special attention was given to proper delivery to the location of infection, i.e., the lungs. Deposition of intranasally administered PvdQ in airways of mice was examined by employing a VivoTag 680XL-labeled PvdQ (PvdQ-VT). Groups of animals were infected with a sublethal dose of *P. aeruginosa* PAO1 and received 50 μL of 1 mg/mL PvdQ-VT intranasally at 0 and 72 h post-bacterial inoculation. The animals were allowed to recover for 5 min after PvdQ-VT administration, followed by *in vivo* imaging as previously described (Tonnis et al., 2014). First, the animal was placed in a Fluorescence Molecular Tomography (FMT, PerkinElmer, Waltham, USA) that permits localization of PvdQ-VT in a three-dimensional visual of the animal. The fluorescence was measured at an excitation wavelength of 660 nm and an emission wavelength of 680 nm. Next, the animal was sacrificed and the isolated lungs were placed on a petri dish, followed by visualization in the *In Vivo* Imaging System (IVIS® Spectrum, PerkinElmer, Waltham, USA). The fluorescence was measured at an excitation wavelength of 675 nm and an emission wavelength of 720 nm. The acquired data from FMT and IVIS were analyzed by TrueQuant™ v3.1 software and Living Image® Software v3.2, respectively. The relative deposition of PvdQ-VT in a certain region of interest was calculated by dividing the fluorescence intensity in the region of interest by intensity of the total area times 100%. Experiments were performed in duplicate, totaling to 6 animals per group.

#### Study 3. efficacy of PvdQ in a mouse pulmonary infection model

The efficacy of PvdQ as a quorum sensing inhibitor was assessed in a lethal (*n* = 6 per group) and a sublethal pulmonary infections with *P. aeruginosa* PAO1. Groups of infected animals received a daily intranasal administration of PvdQ (25 ng/g and 250 ng/g body weight) starting immediately after bacterial inoculation. At 24 and 48 h, mice were sacrificed for quantitative analysis of bacteriology, immune responses and histopathological analysis, unless otherwise stated. Efficacy test in the lethal infection was performed as one experiment (*n* = 6 per group), while experiments for the sublethal infection were performed in triplicate, totaling to 22–23 animals per group.

### Analysis of animal samples

#### Bronchoalveolar lavage

For bronchoalveolar lavage (BAL), the lungs were flushed three times with a total volume of 2 mL PBS supplemented with protease inhibitor (cOmplete™, EDTA-free Protease Inhibitor Cocktail, Roche). Cytospin preparations from 100 μL of unprocessed BAL fluid sample were stained with May Grünwald and Giemsa staining for differential cell counts. Levels of TNF-α and CXCL2 in the cell-free supernatant of BAL fluid (600 rpm slow acceleration, room temperature for 5 min) were measured by ELISA in accordance to the manufacturer's instructions (Duoset, R&D systems).

#### Quantitative bacteriology

Isolated lungs were homogenized in 5 mL PBS using a mechanical homogenizer (IKA-RW15 potter system). Blood, BAL fluid and serial dilution of lung homogenates were plated on the selective media *Pseudomonas* Isolation Agar (PIA) for quantitative bacteriology.

#### Histopathology

Lungs were inflated with cryocompound (Klinipath) and fixed in 4% formaldehyde (Sigma-Aldrich) overnight. Afterwards, the lobes were separated and trimmed prior to paraffin-embedding (Ruehl-Fehlert et al., 2004). Sections of 3–4 μm were stained with haematoxylin and eosin (Sigma-Aldrich). 10–15 areas of the lung sections were scored blindly for peribronchial infiltrates and alveolar involvement at a 40x magnification using an adapted histological scoring system (Table 1; Bayes et al., 2016) that was originally mentioned in Dubin et al. (Dubin and Kolls, 2007).

**Table 1:** Histological scoring system for lung inflammation in infected animals.

**Score**	**Peribronchial infiltrate**	**Alveolar involvement**
0	None	None
1	Low (infiltrate ≤ 4 cells thick)	Low (<25% examined lung with increased cellularity/thickening)
2	Medium (infiltrate 5–10 cells thick)	Medium (25–50% examined lung with increased cellularity/thickening)
3	High (25–50% visualized lumen)	High (>50% examined lung with increased cellularity/thickening)
4	Diffuse (>50% visualized lumen)	

### Statistical analysis

Comparisons between two groups were carried out using Mann Whitney U (non-parametric data). Survival graph was created using the method of Kaplan-Meier, and the comparison of survival between groups was analyzed by the χ^2^-test. Statistical analysis was performed using Graphpad Prism version 5 or SPSS statistics version 25. A probability value (*P*) ≤ 0.05 was considered statistically significant.

## Results

### *In vitro* study of PvdQ

#### Purified PvdQ is active and quenches the virulence of *P. aeruginosa* biosensors in a dose-dependent manner

PvdQ was purified with a yield of 30 mg L^−1^ of cell culture. The protein was >95% pure judged from SDS PAGE with a Coomassie blue staining (Supplementary Figure 1). Purified PvdQ for animal experiments underwent an endotoxin removal step, resulting in a final endotoxin level of 1.6 EU/mg PvdQ, well below the recommended limit for endotoxin in preclinical research (Maylyala and Singh, 2008). The endotoxin removal procedure did not affect the AHL-hydrolyzing activity of PvdQ (Supplementary Figure 2), as shown by the equal degradation of 3-oxo-C12-HSL substrate in both enzymatic reactions.

**Figure 1:**
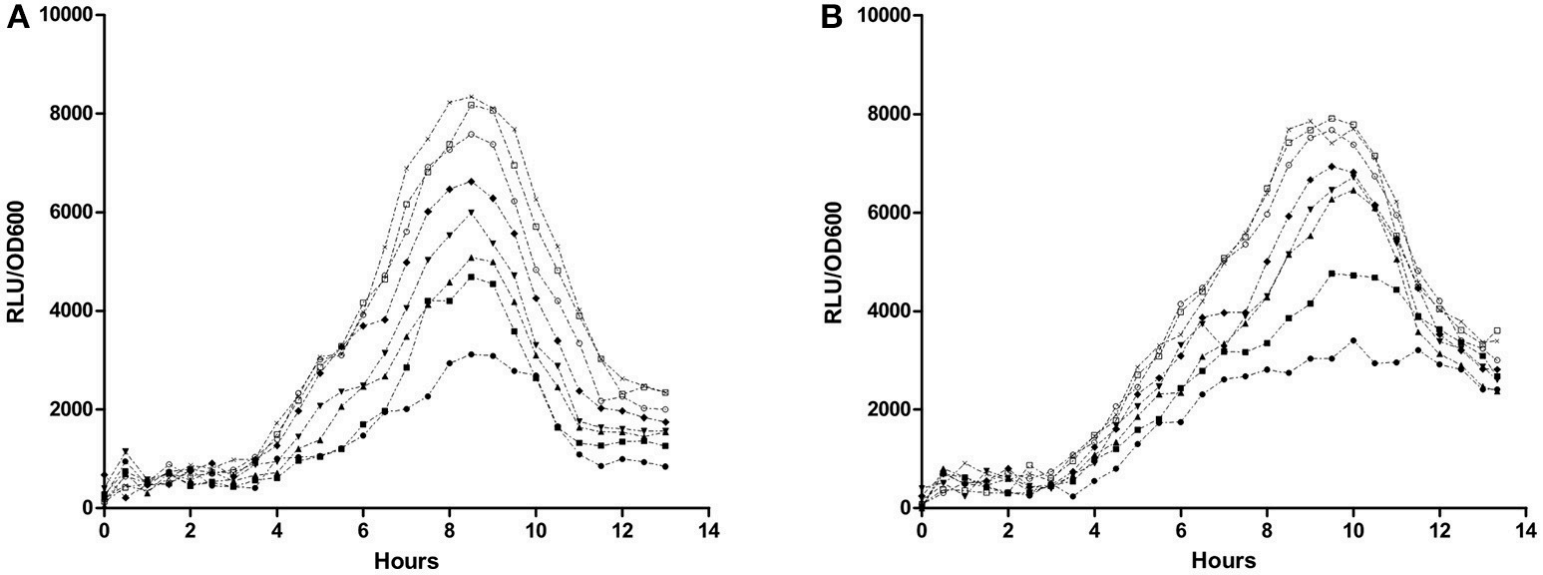
Dose-response curve of PvdQ acylase. Biosensors **(A)** *P. aeruginosa rhlA::lux* and **(B)** *P. aeruginosa lasB::lux* were incubated with various concentration of PvdQ, as follows: 16 μM (●), 8 μM (■), 4 μM (▴), 2 μM (▾), 1 μM (♦), 0.5 μM (○), 0.25 μM (□), and 0 μM (×).

Effectivity of PvdQ in attenuating virulence of *P. aeruginosa* was monitored by employing biosensors with a chromosomal integration of a luciferase gene controlled by the QS-regulated *lasB* promoter or *rhlA* promoter. Emitted luminescence reflects activation of the quorum sensing system, thus the amount of produced light is inversely proportional to the inhibitory strength of PvdQ. The chosen PvdQ doses did not affect growth of the biosensors. Dose-response curves were created by plotting the response of the biosensors as a relative luminescence unit per cell density (Figure 1). The IC50 value could not be calculated since complete signal abolishment was not reached. We could not test a higher concentration of PvdQ to reach a greater signal reduction, because PvdQ precipitates at concentrations above 4 mg/mL.

#### Purified PvdQ does not affect the viability of epithelial cell lines

The toxicity of PvdQ to mammalian cells was assessed *in vitro*, using A549 and H460 human epithelial cell lines. Incubation of cells with up to 10 μM PvdQ for 48 h did not affect the number of viable cells in comparison to control without PvdQ treatment (Supplementary Figure 3), suggesting that PvdQ exhibits minimal to no cytotoxicity toward epithelial cells.

### Validation of the animal model and PvdQ administration procedure

#### The mouse infection model

In principle, the severity of infection in the model depends on the bacterial inoculation dose and the stress level experienced by the animals. In our procedure, the infected animals were receiving a daily administration of PvdQ via intranasal route. Based on pilot experiments, we found that an inoculation dose lower than 10^5^ CFU/lungs resulted in no development of infection, whereas an inoculation dose of 10^6^ CFU/lungs resulted in a severe infection. For the present study we therefore adjusted the inoculation dose to 2.5 × 10^5^ CFU/lungs as a sublethal dose and to twice that amount (5 × 10^5^ CFU/lungs) as a lethal dose. Due to the high discomfort in the lethal infection, the PvdQ distribution study was only performed in the sublethal infection model, while the efficacy of PvdQ was investigated in both levels of infections.

#### Study 1. mouse tolerance of intranasally administered PvdQ

Our studies with mammalian epithelial showed that PvdQ was not toxic to these cells *in vitro*. Based on these results we performed the first part of an *in vivo* study to further ensure safety of intranasally administered PvdQ in mice. Tolerance of non-infected mice to intranasally administered PvdQ was determined with 2 doses of PvdQ (25 and 250 ng/g per animal). Both doses did not induce breathing difficulties, inactivity, poor posture or a drop of body temperature. Mild fluctuations of body weight were observed, with an average of 4% increase or decrease from the initial body weight, which was comparable to the control group receiving sterile beads and a daily intranasal PBS administration. Lungs harvested at 24, 48, and 72 h after the first PvdQ administration showed no macroscopic injury. Histological examination of lungs 72 h after PvdQ administration showed no inflammatory lesions or abnormalities (data not shown).

#### Study 2. *in vivo* imaging to monitor the deposition of intranasally administered PvdQ

**Figure 2:**
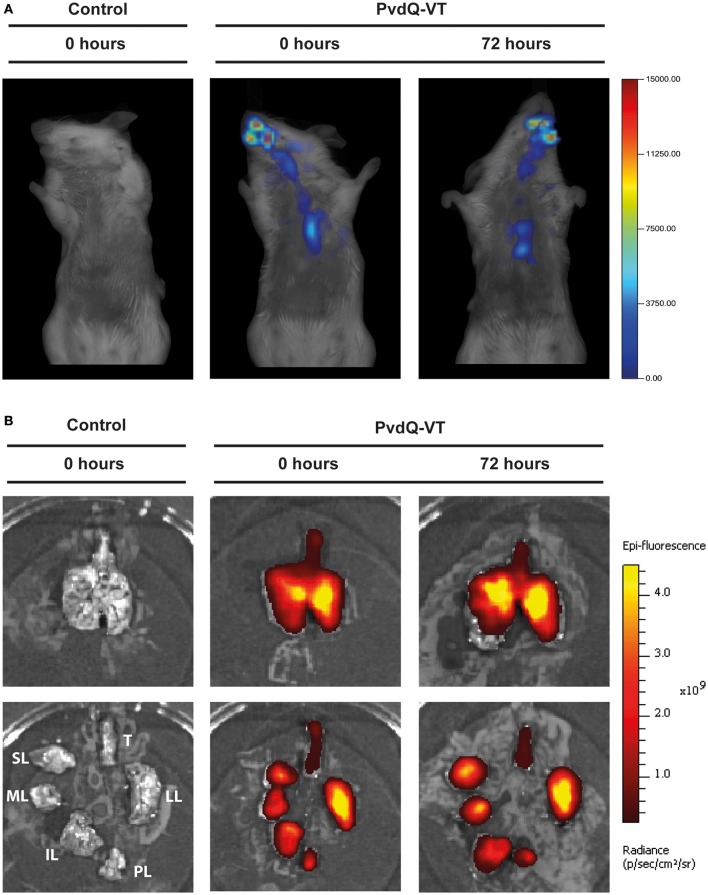
Typical imaging results of animals after intranasal administration of PBS (control, left panel) or PvdQ-VT at different stages of infection (middle and right panels). **(A)** Results from FMT; **(B)** Results from IVIS. Upper panels show intact lungs, while lower panels show trachea and the separated lobes. Legend: Trachea (T), left lung (LL), post-caval lobe (PL), inferior lobe (IL), middle lobe (ML), and superior lobe (SL).

A fluorochrome-tagged PvdQ (PvdQ-VT) was used to ascertain the deposition of the intranasally administered PvdQ-VT in mouse lung tissue. To determine whether infection influences enzyme deposition, PvdQ-VT was intranasally administered to infected animals at different stages of infection (0 and 72 h post-infection) followed by *in vivo* imaging analyses. The Fluorescence Molecular Tomography (FMT) allows a three-dimensional visualization of the whole animal and the typical result is shown in Figure 2A. PvdQ-VT could be traced along the respiratory tract of the animals and 42 ± 7.5% of the delivered dose was deposited in the lungs at 0 h post-infection. At 72 h post-infection, a slightly lower lung deposition was observed (34 ± 5.2%, n.s. compared to 0 h post-infection), and the majority of PvdQ-VT was found in the upper respiratory tract and the head. Afterwards, the lungs were isolated for a more thorough visualization in the *In Vivo* Imaging System (IVIS) and typical data are shown in Figure 2B. PvdQ-VT can be found in all lung lobes with a nearly equal distribution between the right lobes (combined, 47 ± 10.7%) and the left lobe (53 ± 10.7%) at 0 h post-infection. However, at 72 h post-infection, the distribution was shifted with the left lobe containing slightly more (60 ± 8.8%) than the right lobes (40 ± 8.7%).

### Efficacy of PvdQ in a mouse model of pulmonary infection

#### Study 3. (i) treatment with PvdQ results in a longer survival time and higher bacterial clearance during lethal pulmonary infection

**Figure 3:**
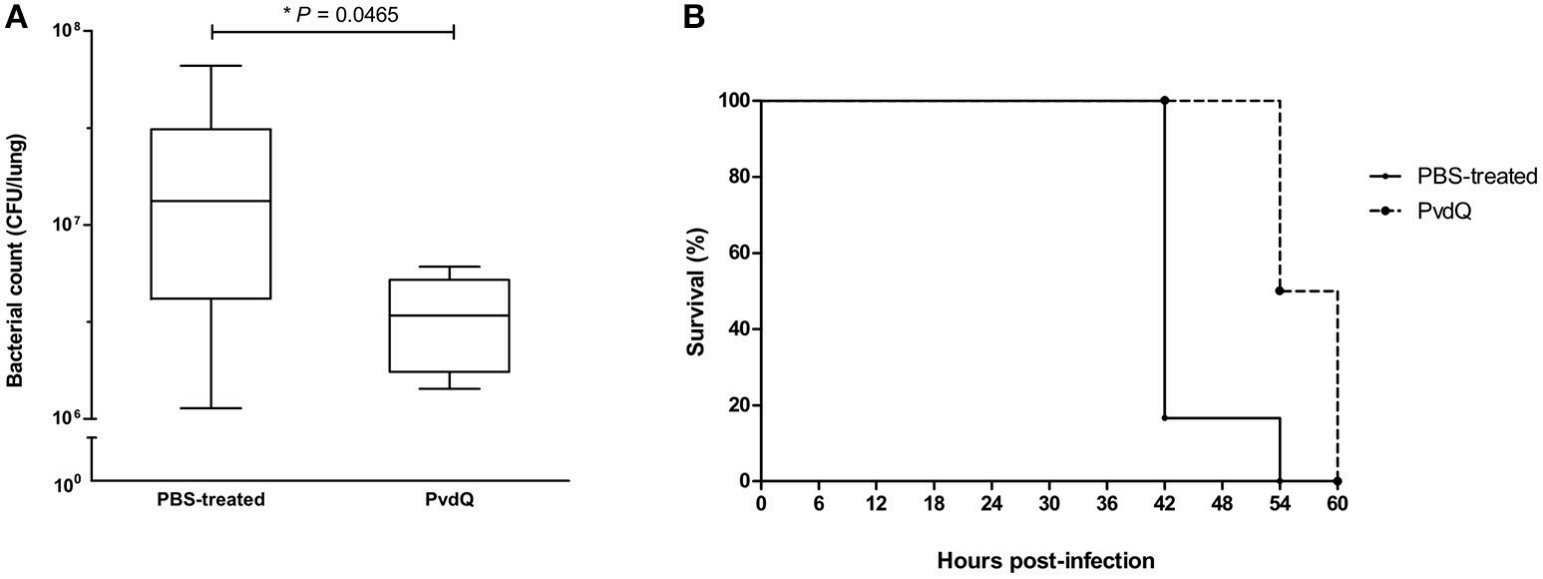
Effects of PvdQ treatment in a lethal *P. aeruginosa* infection mouse model (*n* = 6 animals). **(A)** PvdQ treatment results in a lower load of *P. aeruginosa* in infected animals as compared to PBS-treatment. The bacterial count was obtained 42–60 h post-bacterial-infection. The box and whiskers respectively represent 25–75th percentiles, and range of the data. The horizontal lines represent the median. **(B)** PvdQ-treated animals have a significantly longer survival time than PBS-treated animals.

Having established a pulmonary infection model and the safety of the PvdQ treatment, the next step was to investigate efficacy of PvdQ treatment in this infection model. Treatment of lethally infected animals with PvdQ (25 ng/g) resulted in a 5-fold lower bacterial load for the PvdQ-treated groups than for the PBS-treated group at the end of experiment (*P* = 0.0465, Figure 3A). Furthermore, the PvdQ treatment significantly prolonged the survival time, with a median survival time of 57 h as compared to 42 h in the PBS-treated animals (*P* = 0.004, Figure 3B). The same extent of efficacy was observed with the treatment of 250 ng/g PvdQ (data not shown).

#### Study 3. (ii) PvdQ treatment results in less lung inflammation in a model of sublethal pulmonary infection

Inoculation with a sublethal bacterial dose resulted in a moderately severe infection, with no mortality as a consequence. Using this model, the efficacy of PvdQ treatment was assessed within 48 h post-infection by performing multiple analyses, including quantitative bacteriology, analyses of immune responses and histopathological analysis.

**Figure 4:**
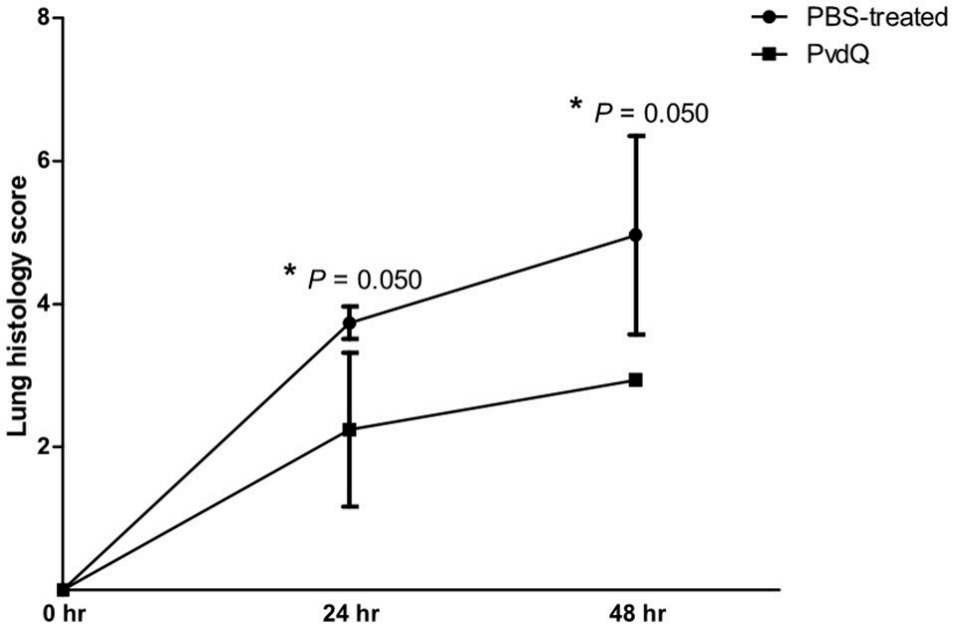
Lung histology scores of lungs of mice treated with PBS (●) or PvdQ (■) at 24 and 48 h post bacterial-infection in a model of sublethal pulmonary infection with *P. aeruginosa*. Three animals were sacrificed from each group at each time point. The graph represents mean and standard deviation.

**Figure 5:**
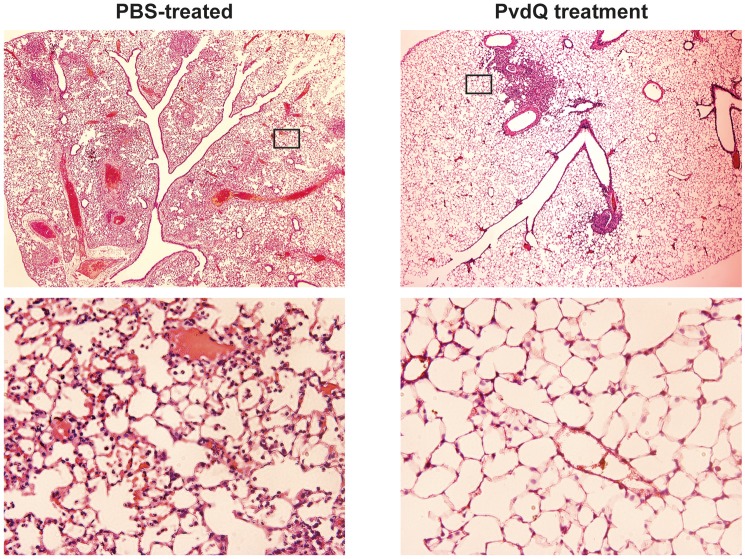
Histological examination of lung tissue of animals infected by sublethal *P. aeruginosa* dose and treated with either PBS or PvdQ at 48 h post-bacterial-infection. Upper panels represent images of H&E stainings at 20x magnification, and the areas marked with rectangular are shown in 200x magnification in the lower panels.

**Figure 6:**
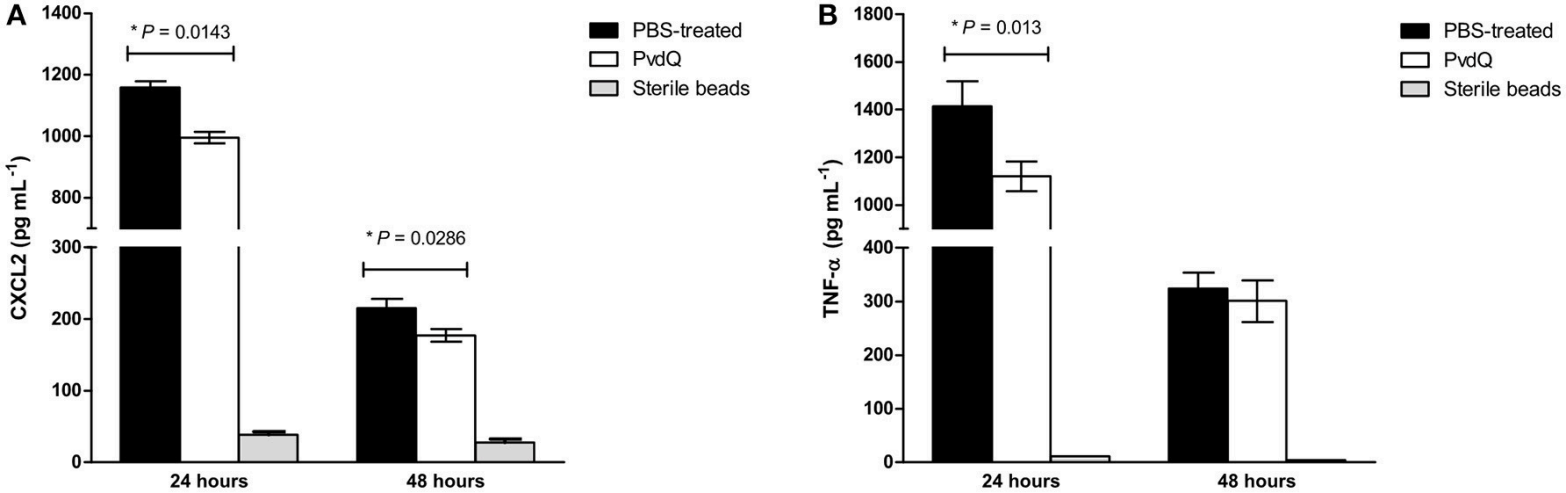
Amounts of **(A)** CXCL2 and **(B)** TNF-α in cell-free BAL fluid from the infected animals treated with PBS (black bars) or PvdQ (white bars). The level of both CXCL2 and TNF-α of PvdQ treatment group was lower in comparison to the PBS-treated group at 24 h post-infection. Three animals were sacrificed from each group at every time point. The bars represent mean and standard deviation.

No significant differences were observed in bacterial load between the PvdQ-treated group and the PBS-treated group at 24 or 48 h post-bacterial-infection (Supplementary Figure 4). No bacteria were found in the blood, spleen or kidney, indicating that the infection was restricted to the lungs. Histopathological analysis of lung tissue showed milder inflammation in the PvdQ-treated group than in the PBS-treated group 24 and 48 h post-infection (Figure 4). Lung tissue of mice treated with PBS showed a higher level of lung injury with diffuse inflammation and swollen alveolar walls, while mice treated with PvdQ showed only small restricted lesions and hardly any alveolar involvement (Figure 5). In line with this finding, the levels of CXCL2 and TNF-α in BAL fluid of PvdQ-treated mice were significantly lower compared to PBS-treated mice at 24 h post-infection. At 48 h post-infection the levels of immune response indicators were similar between both groups and almost back to the levels found in non-infected animals (Figure 6). The total number of inflammatory cells in BAL fluid of PvdQ and PBS-treated animals was higher as compared to non-infected animals (Supplementary Figure 5A), but no differences were seen between PBS- and PvdQ-treated animals. In addition, the number of neutrophils in BAL fluid was assessed, but again no differences were seen between PBS and PvdQ treatment (Supplementary Figure 5B). The same extent of efficacy was observed with the treatment of 250 ng/g PvdQ (data not shown).

## Discussion

*Pseudomonas aeruginosa* infection is a growing problem in the healthcare, as well as being the predominant pathogen in pulmonary infections of cystic fibrosis patients. Multiple factors are contributing to the tenacity of *P. aeruginosa* as a human pathogen, including its remarkable adaptability that allows this bacterium to establish a successful infection and to escape antibiotic treatments. In the wake of the antibiotic resistance problem, relatively much attention has been given to the study of quorum sensing inhibitors (QSIs) as novel antibacterial candidates (Kalia, 2013; LaSarre and Federle, 2013; Fetzner, 2014). They fall into the category of antivirulence drugs that generate less selective pressure for evoking resistance in comparison to conventional antibiotics. AHL-hydrolyzing enzymes prevent accumulation of AHLs and the QQ effects by some of these enzymes are evident in infection models. Nevertheless, the number of the documented studies in mammals is relatively small, given the abundance of the characterized QQ enzymes. The first study in a pulmonary infection model was conducted by Migiyama and colleagues, showing that a *P. aeruginosa* strain overexpressing AiiM lactonase is less virulent than the wild-type (Migiyama et al., 2013). This finding was followed by a report from Hraiech and colleagues who employed a purified *Sso*Pox-I lactonase as a therapeutic agent in a lethal *P. aeruginosa* pulmonary infection model in rats (Hraiech et al., 2014). The purified *Sso*Pox-I lactonase was administered through the intubation of the exposed trachea and could reduce the mortality of the infected animals. Although these studies excellently demonstrated the therapeutic value of AHL-hydrolyzing enzymes, there is yet no study using a non-invasive administration route of the enzymes that closely mimics the possible drug administration route in human. In the present study, we have shown that PvdQ is well-tolerated by human lung epithelial cell lines, indicating that PvdQ has minimal or no cytotoxic effects on human cells. Furthermore, intranasally administered PvdQ acylase is well-tolerated and distributes well in lung tissue of mice, even during infection. Most importantly, intranasally administered PvdQ acylase alleviates *P. aeruginosa* pulmonary infection in mice, which may lead to faster resolution of the infection.

Prior studies have confirmed that supplementation of PvdQ to cultures of *P. aeruginosa* inhibits accumulation of 3-oxo-C12-HSL and in turn blocks production of elastase and pyocyanin (Sio et al., 2006). Furthermore, PvdQ showed a therapeutic effect in a *C. elegans* model of *P. aeruginosa* infection (Papaioannou et al., 2009). In order to test the preclinical efficacy of PvdQ in a more relevant animal model, we developed a mouse model combining the *P. aeruginosa* pulmonary infection with an administration procedure that can be translated to the human situation. A pulmonary infection model is very challenging to be developed in mouse (van Heeckeren and Schluchter, 2002), even more so when the infection is combined with a topical drug administration method. Lung-targeted delivery systems of large molecules in animal can be performed via pulmonary inhalation by different procedures, such as passive inhalation of aerosolized drugs (whole body, head-only, or nose-only exposure system), direct intratracheal administration or intranasal administration (Fernandes and Vanbever, 2009). Arguably, among these methods, a nose-only aerosol system would be of highest resemblance to that of in human, such as the inhalation of aerosolized DNAse Pulmozyme® for cystic fibrosis patients. However, the major drawback of this method is the requirement of highly accurate instruments, an ample amount of drugs, and a long exposure time (30–45 min) that could subject the infected animals to high level of stress. Intranasal delivery is one of the most common, and the least intrusive method for this purpose (Southam et al., 2002; Fernandes and Vanbever, 2009), hence it was chosen as the drug administration procedure in our experiment. Despite its simplicity, the downside of this intranasal delivery is the difficulty in controlling the dose deposition efficiency, because the drugs have to travel all the way through the upper respiratory tract before finally reaching the lungs.

Lung deposition efficiency from intranasal administration of fluorochrome-tagged PvdQ (PvdQ-VT) at 0 h post-bacterial-inoculation is in concordance to the study of Eyles and colleagues. They observed 48 ± 12.1% of radiolabeled 7-μm-diameter polymer microspheres in the healthy mouse lungs after an intranasal challenge (Eyles et al., 1999). In our study, the reduced lung deposition efficiency at the later stage of infection might be a repercussion of lung function deterioration caused by bacterial infection, such as a decrease of the inspired air volume as seen in other studies (Wölbeling et al., 2010, 2011). At 72 h post-bacterial infection, a shift of deposition toward the left lobe was observed. This finding is presumably related to the structural changes experienced by each lobe. However, to explain specific regional functions of the lungs, further research with a more elaborate function-related physiology study (e.g., determination of airspace diameters) is required.

The efficacy of PvdQ was assessed in mouse models with different levels of infection lethality. PvdQ administered via an intranasal route during lethal infection resulted in a lower bacterial load in the lungs, demonstrating a role of PvdQ in promoting bacterial clearance (Figure 3). Since the delivered PvdQ is a sub-MIC dose that did not affect bacterial growth *in vitro* and in a *C. elegans* infection model, we strongly believe that PvdQ does not clear the infection itself but is helping the immune system by disarming the bacteria in the mouse infection model. As a result of the lowered bacterial load, survival time of PvdQ treatment group was increased, in agreement with other murine studies of AHL-lactonases AiiM (Migiyama et al., 2013) and *Sso*Pox-I (Hraiech et al., 2014). In addition, our results also corroborate with the findings from animal studies of small molecule QSIs, such as furanone, patulin and garlic extracts (Wu et al., 2004; Bjarnsholt et al., 2005; Rasmussen et al., 2005). However, some of these QSIs such as patulin and furanone are known to be toxic for mammals (Hentzer and Givskov, 2003; Puel et al., 2010). In addition, the small molecule QSIs having intracellular targets are prone to development of resistance via upregulated efflux pumps (García-Contreras et al., 2013). The median survival after PvdQ treatment is longer than shown for the group of animals receiving a deferred *Sso*Pox-I lactonase treatment (45 h) in the study of Hraiech and colleagues (Hraiech et al., 2014). Direct comparisons with the group receiving an immediate treatment is not possible because the median survival cannot be calculated from their data as they stopped their observation after 50 h post-bacterial infection. The fact that our mice eventually were still dying even though the bacterial load is lower, may be related to an overwhelming inflammatory response. The high bacterial load may induce an excess of inflammatory responses that cannot be counteracted by PvdQ disarming virulence factors anymore.

In order to perform an extensive analysis of immune responses, we extended our study with a more thorough examination during a sublethal infection. The experimental setup was similar to that of the lethal infection, but with a smaller bacterial inoculum. Consequently, the sublethal infection was milder and the defense mechanisms themselves could clear the infection, resulting in a 1,000-fold lower bacterial CFU in comparison to the lethal infection. The treatment with PvdQ in the sublethal *P. aeruginosa* infection did not lead to a lower bacterial count in comparison to the PBS-treated group (Supplementary Figure 4), but resulted in less lung inflammation (Figures 4, 5) as well as lower levels of CXCL2 and TNF-α (Figure 6) suggesting that virulence has been suppressed. High levels of proinflammatory cytokines are observed during bacterial infection in CF patients, including IL-8 (a human analog of CXCL2 in mouse) and TNF-α (Richman-Eisenstat, 1996). The high levels of IL-8 and TNF-α in the sputum positively correlate with clinical symptoms of deterioration in CF patients and antibiotic treatment resulted in lower levels of both cytokines (Karpati et al., 2000; Colombo et al., 2005). Numerous bacterial virulence factors are known to activate innate immune responses, while others are responsible for tissue damage during infection. This includes 3-oxo-C12-HSL that is not only a potent chemoattractant of neutrophils (Karlsson et al., 2012) but also can induce an inflammatory response by macrophages (Telford et al., 1998; Thomas et al., 2006). Many QS-regulated virulence determinants are known for their tissue destructive properties, among them is elastase that hydrolyses protein elastin of lung tissue (Van Delden and Iglewski, 1998). Our observations in the sublethal infection model indicate that PvdQ treatment may reduce lung inflammation by preventing the accumulation of 3-oxo-C12-HSL and thereby diminishing the production of virulence factors that contribute to lung injury. We observed no difference in the number of inflammatory cells in BAL fluid from the PBS treatment group, even though a considerably higher amount of cells was found at the epithelial tissue of the PBS-treated group (Figure 5). Extracellular factors of *P. aeruginosa* such as 3-oxo-C12-HSL (Tateda et al., 2003), rhamnolipid (Jensen et al., 2007), and pyocyanin (Allen et al., 2005) potentially induced apoptosis of the neutrophils that migrated to the alveolar space, reducing the number of cells in BAL fluid. The dose of 25 ng/g is presumably sufficient to fully hydrolyze extracellular AHLs in the lungs. Hence, increasing the PvdQ dose further did not improve the therapeutic efficacy in both lethal and sublethal infections.

Taken together, our study shows that the intranasally administered PvdQ acylase can act as a therapeutic QQ enzyme to attenuate *P. aeruginosa* in a mouse pulmonary infection model. The inhibition of *P. aeruginosa* virulence clearly contributed to bacterial clearance and an improved condition of the lungs. Hence, PvdQ by itself can be a potential candidate as a part of the treatment of pulmonary infection. Increasing the shelf-life of PvdQ is achievable by formulating it into a dry powder that is suitable for inhalation (Wahjudi et al., 2012). Another interesting approach is to employ PvdQ in the combination therapy to increase the efficacy of conventional antibiotics. Therefore, in the future studies, expanding the therapeutic application of PvdQ would be of high interest.

## Author contributions

WQ is the principal investigator who initiated the project of quorum quenching. All authors contributed in designing the experiments. PU and RS performed the experiments and analyzed the data. The manuscript was written by PU and was carefully revised by BM and WQ.

### Conflict of interest statement

The authors declare that the research was conducted in the absence of any commercial or financial relationships that could be construed as a potential conflict of interest.
