# Supplementary material for: PvdQ Quorum Quenching Acylase Attenuates Pseudomonas aeruginosa Virulence in a Mouse Model of Pulmonary Infection
Source: Front Cell Infect Microbiol. 2018 Apr 26;8:119. doi: 10.3389/fcimb.2018.00119 (PMC5932173; doi:10.3389/fcimb.2018.00119)
Supplement: Supplementary file 1 [file Image_1.PDF]

Supplementary Material

PvdQ quorum quenching acylase attenuates *Pseudomonas aeruginosa* virulence in a mouse model of pulmonary infection

Putri Dwi Utari, Rita Setroikromo, Barbro N. Melgert, Wim J. Quax

*** Correspondence:** Wim J. Quax: w.j.quax@rug.nl

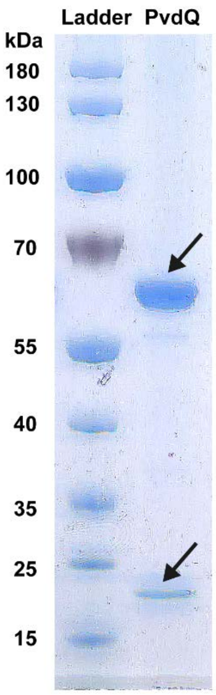

Supplementary Figure 1. Purified PvdQ on a SDS PAGE gel. The first lane shows the protein ladder (Prestained Pageruler, Thermo Fisher). PvdQ appears as two subunits: the small subunit alpha and the large subunit beta (indicated with the arrows).
