# Supplementary material for: PvdQ Quorum Quenching Acylase Attenuates Pseudomonas aeruginosa Virulence in a Mouse Model of Pulmonary Infection
Source: Front Cell Infect Microbiol. 2018 Apr 26;8:119. doi: 10.3389/fcimb.2018.00119 (PMC5932173; doi:10.3389/fcimb.2018.00119)
Supplement: Supplementary file 2 [file Image_2.PDF]

Supplementary Material

PvdQ quorum quenching acylase attenuates *Pseudomonas aeruginosa* virulence in a mouse model of pulmonary infection

Putri Dwi Utari, Rita Setroikromo, Barbro N. Melgert, Wim J. Quax

*** Correspondence:** Wim J. Quax: w.j.quax@rug.nl

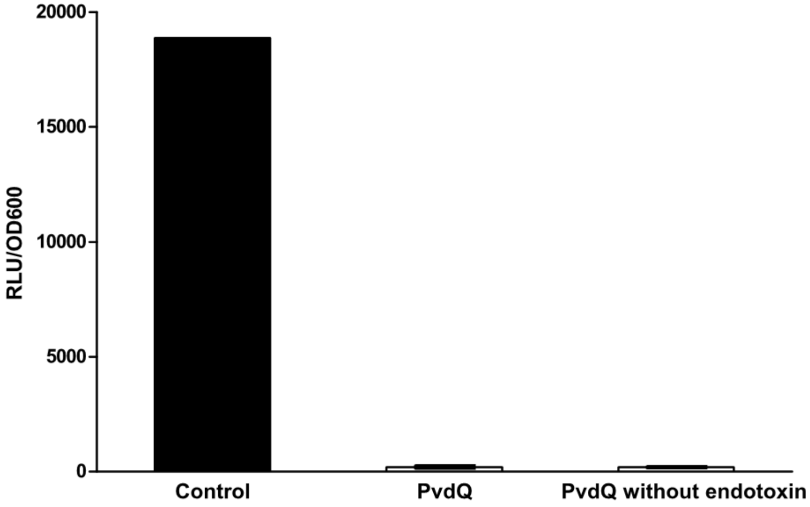

Supplementary Figure 2. Bioactivity assay of PvdQ acylase in hydrolyzing 3-oxo-C12-HSL substrate, before (PvdQ) and after endotoxin removal treatment (PvdQ-ET). The emitted light is proportional to the presence of 3-oxo-C12-HSL. Heat-inactivated PvdQ is used in the control reaction. The result is presented as a mean of 6 individual wells and standard deviation (SD).
