# Supplementary material for: PvdQ Quorum Quenching Acylase Attenuates Pseudomonas aeruginosa Virulence in a Mouse Model of Pulmonary Infection
Source: Front Cell Infect Microbiol. 2018 Apr 26;8:119. doi: 10.3389/fcimb.2018.00119 (PMC5932173; doi:10.3389/fcimb.2018.00119)
Supplement: Supplementary file 3 [file Image_3.PDF]

Supplementary Material

PvdQ quorum quenching acylase attenuates *Pseudomonas aeruginosa* virulence in a mouse model of pulmonary infection

Putri Dwi Utari, Rita Setroikromo, Barbro N. Melgert, Wim J. Quax

*** Correspondence:** Wim J. Quax: w.j.quax@rug.nl

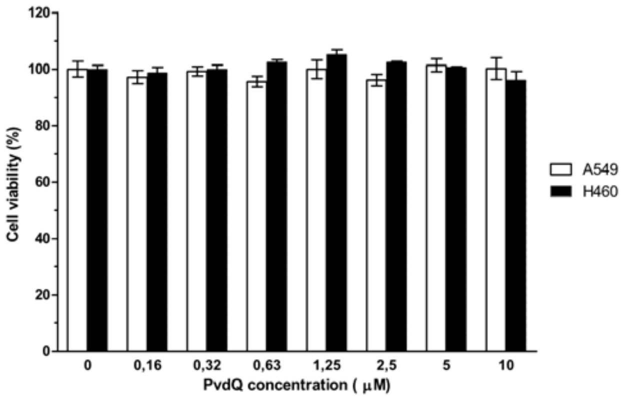

Supplementary Figure 3. Viability of human epithelial cell lines A549 (white bars) and H460 (black bars) after 48 hours incubation with PvdQ. Cell viability is shown as percentage value relative to the control (0 μM of PvdQ).
