# Supplementary material for: PvdQ Quorum Quenching Acylase Attenuates Pseudomonas aeruginosa Virulence in a Mouse Model of Pulmonary Infection
Source: Front Cell Infect Microbiol. 2018 Apr 26;8:119. doi: 10.3389/fcimb.2018.00119 (PMC5932173; doi:10.3389/fcimb.2018.00119)
Supplement: Supplementary file 4 [file Image_4.PDF]

frontiers

Supplementary Material

PvdQ quorum quenching acylase attenuates *Pseudomonas aeruginosa* virulence in a mouse model of pulmonary infection

Putri Dwi Utari, Rita Setroikromo, Barbro N. Melgert, Wim J. Quax

*** Correspondence:** Wim J. Quax: w.j.quax@rug.nl

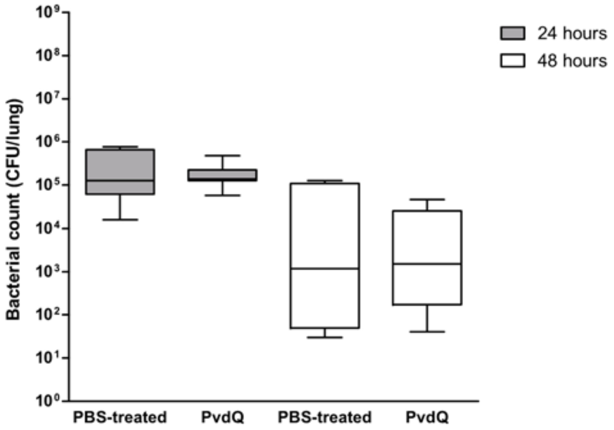

Supplementary Figure 4. Bacterial loads in lungs of mice treated with PBS or PvdQ 24 hours (grey bars) and 48 hours (white bars) post-bacterial infection in a model of sublethal pulmonary infection of *P. aeruginosa*. Seven animals were sacrificed from each group at each time point. The box and whiskers respectively represent 25th to 75th percentiles, and range of the data. The horizontal lines represent the median.
