# Supplementary material for: PvdQ Quorum Quenching Acylase Attenuates Pseudomonas aeruginosa Virulence in a Mouse Model of Pulmonary Infection
Source: Front Cell Infect Microbiol. 2018 Apr 26;8:119. doi: 10.3389/fcimb.2018.00119 (PMC5932173; doi:10.3389/fcimb.2018.00119)
Supplement: Supplementary file 5 [file Image_5.pdf]

Supplementary Material

PvdQ quorum quenching acylase attenuates *Pseudomonas aeruginosa* virulence in a mouse model of pulmonary infection

Putri Dwi Utari, Rita Setroikromo, Barbro N. Melgert, Wim J. Quax

*** Correspondence:** Wim J. Quax: w.j.quax@rug.nl

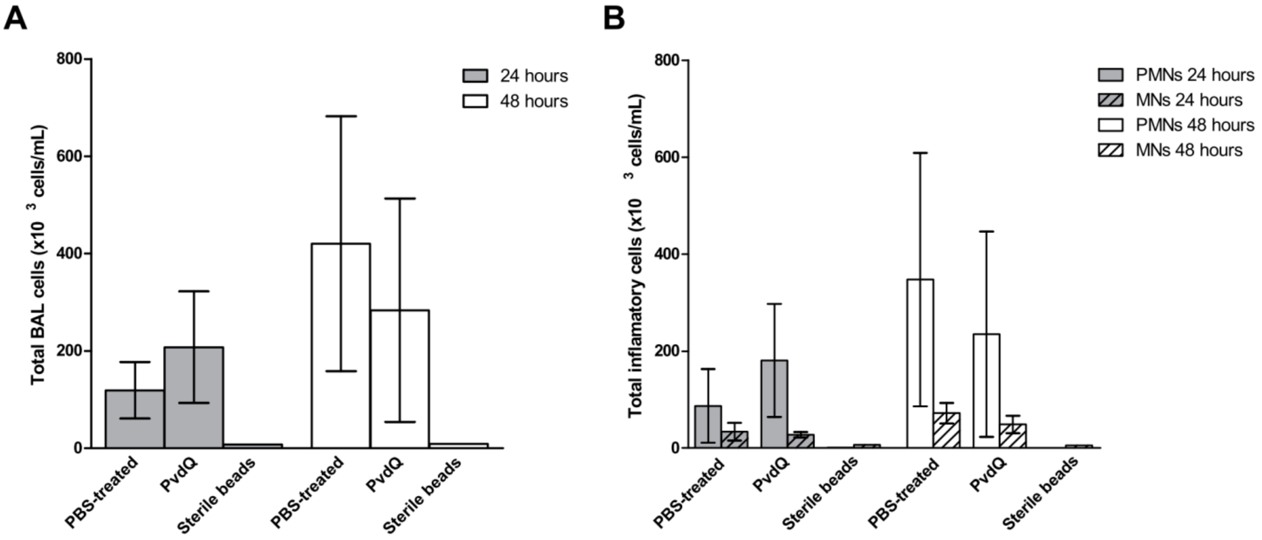

Supplementary Figure 5. BAL fluid analysis from animals treated with PBS or PvdQ 24 hours (grey bars) and 48 hours (white bars) post-bacterial infection in a model of sublethal pulmonary infection of *P. aeruginosa*. **A.** Total cell counts, and **B.** Polymorphonuclear leukocytes (PMNs, neutrophils) and Mononuclear leukocytes (MNs) cells in BAL fluid. Three animals were sacrificed from each group at every time point. The bars represent mean and standard deviation.
